# A Novel Drinking Category Detection Method Based on Wireless Signals and Artificial Neural Network

**DOI:** 10.3390/e24111700

**Published:** 2022-11-21

**Authors:** Jie Zhang, Zhongmin Wang, Kexin Zhou, Ruohan Bai

**Affiliations:** 1 School of Computer Science and Technology, Xi’an University of Posts and Telecommunications, Xi’an 710121, China; 2 Shaanxi Key Laboratory of Network Data Analysis and Intelligent Processing, Xi’an 710121, China; 3 Xi’an Key Laboratory of Big Data and Intelligent Computing, Xi’an 710121, China; 4 School of Information Science and Technology, NorthWest University, Xi’an 701127, China

**Keywords:** drinking category detection, wireless signals, artificial neural network

## Abstract

With the continuous improvement of people’s health awareness and the continuous progress of scientific research, consumers have higher requirements for the quality of drinking. Compared with high-sugar-concentrated juice, consumers are more willing to accept healthy and original Not From Concentrated (NFC) juice and packaged drinking water. At the same time, drinking category detection can be used for vending machine self-checkout. However, the current drinking category systems rely on special equipment, which require professional operation, and also rely on signals that are not widely used, such as radar. This paper introduces a novel drinking category detection method based on wireless signals and artificial neural network (ANN). Unlike past work, our design relies on WiFi signals that are widely used in life. The intuition is that when the wireless signals propagate through the detected target, the signals arrive at the receiver through multiple paths and different drinking categories will result in distinct multipath propagation, which can be leveraged to detect the drinking category. We capture the WiFi signals of detected drinking using wireless devices; then, we calculate channel state information (CSI), perform noise removal and feature extraction, and apply ANN for drinking category detection. Results demonstrate that our design has high accuracy in detecting drinking category.

## 1. Introduction

With the development of people’s living standards and the gradual enhancement of health awareness, the consumption demand for beverages is also rising. At the same time, it is beginning to show a diversified trend. People no longer focus on the function of thirst quenching, and prefer natural, low sugar, and healthy drinks [1,2]. In addition to providing water and other nutrients necessary for human life, different kinds of drinks play different roles in the human body. Some drinks have the function of dietotherapy and healthcare, and some drinks have the function of regulating body function [3].

According to research from the United States, drinking one or two glasses of beer a day can help bones be healthier [4]. For women, drinking one or two glasses of wine a day has the same effect [5,6]. However, researchers warn that the important thing is to drink in moderation; when things go too far, they will lead to osteoporosis. Many applications, however, would benefit from knowing the exact drinking category. For example, safety checks in public transport and adulterant identification. Security personnel need to know the category of liquid accurately to avoid dangerous liquids such as flammables and explosives being brought into public transportation and affecting people’s safety. At present, there are many counterfeit products on the market to confuse consumers, such as counterfeiting luxury perfumes, making milk with water and additives, making liquor with methanol, and counterfeit and shoddy medicines. Among them, methanol and counterfeit drugs are harmful to the human body.

Currently, many industries are solving this problem with some instruments. For example, drinking categories can be detected and drinking concentrations analyzed by conventional viscosity testing instruments [7,8,9,10]. However, these instruments are expensive and invasive. In addition, because the instruments need to be inserted into the probe for detection, it will contaminate the drinking.

In recent years, non-contact drinking category detection methods based on wireless signals such as radio frequency (RF) signals [11,12,13], Ultra-wideband (UWB) signals [14], etc. have been researched. For example, LiquID [14] uses UWB signals to measure the permittivity of liquids to detect drinking categories. However, these methods commonly rely on expensive equipment and these signals are not widely popularized in our daily life.

This paper introduces a novel drinking category detection method based on wireless signals and an artificial neural network that leverages WiFi signals to identify drinking categories, which are inexpensive and ubiquitous with WiFi devices. The intuition of our design is that when the WiFi signals propagate in different drinking, the signals will have different attenuation [15], which is the basis of the detection method based on the WiFi signals, which can be used as fingerprints to identify different drinking categories.

Our design employs a transmitter and a receiver, which are commercial off-the-shelf (COTS) devices, for transmitting and receiving wireless signals, respectively. The wireless signals are transmitted from the transmitter to the target to be tested and then propagated to the receiver through multiple paths. Different drinking categories have different multipath profiles, also known as CSI measurements, which can be leveraged to detect drinking categories.

One challenge is the noise in the CSI measurements, resulting in lower detection accuracy for drinking categories. Since WiFi devices are susceptible to interference, CSI measurements are noisy even in static environments without human activity. Moreover, because conventional denoising methods are only suitable for low-density noise, and the noise density in CSI measurements is higher, conventional denoising methods do not perform well in removing noises. To overcome this challenge, our design leverages a principal component analysis (PCA)-based CSI denoising method.

Another challenging problem is the need to extract effective features from the received wireless signals for drinking category detection. Different materials will change the propagation paths of wireless signals, resulting in different multipath effects. The detection method based on wireless signals detects the drinking categories by capturing multipath profiles of different drinks. Therefore, feature extraction is the basis of this method, and the effective features will improve the overall performance of our design. To remove the limitations, we extract fifteen time–frequency domain statistical features and then use ANN to detect and classify drinking categories.

**Summary of Results:** We built a model of our design using COST devices as a transceiver and evaluated it with six common drinking categories. Our experimental results show that our design can achieve high detection accuracy.

**Contributions:** Our design makes the following contributions:

1. It presents a novel drinking category detection method based on wireless signals and an artificial neural network. As a result, our design has high detection accuracy and high classification precision.
2. It demonstrates that ANN performs well in drinking category detection compared with traditional machine learning methods.

## 2. Materials and Methods

### 2.1. Sample Preparation

The beverage is a liquid for human or livestock drinking; it is a product with quantitative packaging for direct drinking or mixing or brewing with water in a certain proportion, where the ethanol content (mass content) does not exceed 0.5%. According to the general classification rules for beverages, beverages can be divided into 11 categories: packaged drinking water, fruit and vegetable juice, carbonated beverages, protein beverages, special purpose beverages, flavor beverages, tea beverages, coffee beverages, plant beverages, solid beverages, and other beverages.

The classification includes the following:

- Carbonated beverages (soft drinks) refer to drinks filled with carbon dioxide gas under certain conditions, generally including Coke, Sprite, soda, etc.
- Fruit and vegetable juice drinks refer to fruit and vegetable juice obtained directly from refrigerated or fresh vegetables and fruits without the addition of any foreign substances, and are made from fruit and vegetable juice with water, sugar, acid, or spices. Generally includes fruit juice, fresh juice, vegetable juice, mixed fruit and vegetable juice, etc.
- Energy drinks (functional drinks) refer to a beverage that regulates human function to a certain degree by changing the composition and nutritional content percentage of the drink. According to energy drink categorization based on relevant references [16], they are considered functional drinks in a broad sense including polysaccharide beverages, vitamin beverages, mineral beverages, sports beverages, probiotic beverages, low-energy beverages, and other beverages with healthcare functions.
- Tea drinks refer to tea products made by soaking the tea in water, extracting, filtering, or clarifying, and/or by adding water, sugar, sour, food flavors, and fruit juices into the tea soup. Generally includes green tea, black tea, oolong tea, wheat tea, herbal tea, fruit tea, etc.
- Milk beverages refer to the products made from fresh milk or dairy products after fermentation or without fermentation, generally including milk, yogurt, milk tea, etc.
- Coffee drinks are made from roasted coffee beans. Generally includes coffee.

In this paper, we investigate six kinds of drinks in all.

### 2.2. Preliminary about Wireless Sensing

Wireless sensing technology refers to the non-contact sensing technology for people and the environment through universal wireless signals, such as electromagnetic waves [17], light waves [18], and sound waves [19]. The technology has broad application prospects in the Internet of Things, artificial intelligence, healthcare, and national defense.

**Figure 1:**
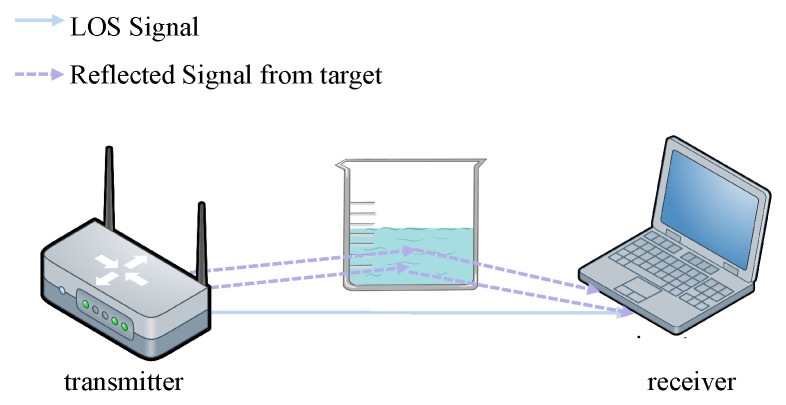
The rationale of wireless-signal-based drinking category detection. When different drinking categories are detected, the multipath effect causes different distortions that may be used as fingerprints to detect drinking category.

Taking the RF signal as an example, the principle of wireless sensing is that the wireless signal generated by the transmitter has physical phenomena such as direct reflection and scattering in the propagation process, thus forming multiple propagation paths, as shown in Figure 1. The multipath signal received at the receiver carries the information reflecting the signal propagation space. Wireless sensing technology obtains the characteristics of signal propagation space by analyzing the changes of wireless signals in the propagation process so as to realize scene sensing.

Compared with current sensing technology [20,21,22,23], it has the three following advantages: (1) sensorless, it is no longer necessary to deploy special sensors to sense people and environment, which is different from wireless sensor networks in which sensors are responsible for sensing and wireless signals are responsible for communication; (2) wireless, no need to deploy wired lines for communications and sensors; (3) contactless, compared with various wearable smart devices, users do not need to wear any devices.

### 2.3. Channel State Information

CSI describes how the WiFi signals propagate from the transmitter to the receiver [24], reflecting the impacts of signal propagation, such as scattering, attenuation, etc.

When the deployed device has *n* transmit antennas and *m* receive antennas, the system will receive *m* × *n* × *s* subcarriers at the receiver, where *s* is the number of subcarriers in each channel. In addition, the measured channel frequency response H(f,t) can be expressed by the following formula [25]:

(1) $$H\left(f,t\right)=\frac{Y(f,t)}{X(f,t)}$$

where X(f,t) and Y(f,t) are the transmit signals and the received signals, and *f* and *t* are frequency and time, respectively.

Currently, there are two key methods for drinking category detection based on wireless signals, including received signal strength (RSS) and CSI.

The WiFi signals propagate through the target to the receiver via reflection, refraction, attenuation, etc., resulting in wireless signals distortion, which is known as the multipath effect. RSS is sensitive to the environment and is vulnerable to multipath propagation, which affects detection accuracy [26]. Furthermore, RSS-based detection methods do not give fine-grained channel data.

CSI describes how the physical environment affects the wireless signals [24]. Furthermore, CSI may provide fine-grained information about WiFi signal propagation—such as time delays, amplitude attenuation, and so on—of multipaths on each subcarrier, which can expose information about signal propagation.

Compared with RSS, CSI can obtain more fine-grained information and higher accuracy [27]; so, our design chooses the drinking category detection method based on CSI.

**Figure 2:**
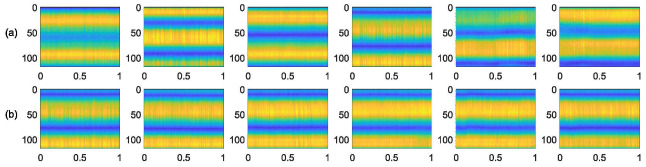
The CSI magnitude images of six detected drinks. The X-axis is the time, the Y-axis is the subcarrier, and the color represents the size of the magnitude. (**a**) The CSI magnitudes for different categories of drinking. (**b**) The CSI magnitudes collected multiple times for the same category of drinking.

The WiFi-signal-based drinking category detection method relies on similar CIR measurements. To analyze whether WiFi signals can detect drinking categories, we plot the CSI magnitude images for different drinks in the same environment and the same drinks collected multiple times, as shown in Figure 2. From Figure 2, we can see that different drinks have different CSI magnitudes, which can be used as a fingerprint for drinking category detection. Meanwhile, the CSI magnitudes of the same drinking are similar, which proves the stability of our design.

## 3. Drinking Category Detection

**Figure 3:**
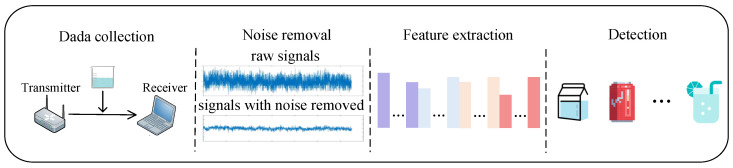
Framework of the drinking category detection method.

Figure 3 shows the framework of the detection method, which includes data collection and noise removal, feature extraction, and detection in three main phases.

### 3.1. Data Collection and Noise Removal

**Data Collection.** Our design uses two wireless devices to collect CSI measurements at the receiver end of the wireless link, one as a transmitter and the other as a receiver. Current CSI-based detection methods collect CSI measurements using the PicoScenes tool [28,29], which uses the IWL 5300 NIC (Network Interface Card). Besides, the transmitter is a router and the receiver is a personal computer (PC) or laptop with NICs. For our design, we used an IWL 5300 NIC with two antennas as the receiver and a router as the transmitter. The sequence of CSI time series for each subcarrier for a given pair of transmitting and receiving antennas is called a CSI stream. Our design uses the PicoScenes tool to collect data; since our design sends a packet per millisecond, the system receives 1000 packets per second. In addition, after analysis by PicoScenes MATLAB Toolbox [30], 117 subcarriers are received.

**Noise Removal.** For the fluctuation of the collected CSI measurements due to the interference of factors such as internal CSI reference levels, transmission rates, and transmit power levels, CSI measurements frequently contain noise in the time domain and frequency domain. Therefore, the collected CSI measurements need to be denoised for further feature extraction and drinking category detection. In this paper, our design uses Principal Component Analysis (PCA) to remove noise [31,32,33,34], as detailed below.

Generally, the denoising steps of PCA include preprocessing, correlation estimation, eigendecomposition, and movement signal reconstruction. First, 1-second data are intercepted for each CSI stream and the average is calculated as a constant offset for each CSI stream, which is the average CSI amplitude. After that, the static path components in each CSI stream are removed by subtracting the corresponding offset from each stream. Next, the remaining CSI streams are formed into a matrix of CH. Then, we calculate the correlation matrix, denoted CHT×CH. The dimension of the matrix is n×n, where *n* is the CSI stream size and n=117. Next, we decompose the features of the correlation matrix to calculate its eigenvectors. Finally, we reconstruct the movement signal. We construct the principal components using the following equation:

(2) pi=CH×ei

where ei and pi are separately the ith eigenvector and the ith principal component.

The first principal component p1 contains noise and CSI reflected back by the target. The CSI measurements are also included in other principal components [31]; so, we discard p1 and retain the remaining 30 principal components as denoised CSI measurements for feature extraction.

**Figure 4:**
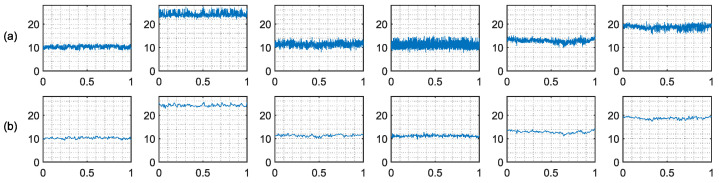
The CSI measurements of different drinking categories. The X-axis is the time and the Y-axis is the magnitude. (**a**) Before denoising. (**b**) After denoising.

We plot the CSI measurements of different drinking categories before and after denoising, as shown in Figure 4. From Figure 4, we can find that there are differences in CSI measurements for different drinking before and after denoising, which can be used as a fingerprint for drinking category detection. Moreover, the CSI measurements after denoising are smoother, which proves that the noise has been removed.

### 3.2. Feature Extraction

Time domain statistics features [35,36] are extracted, such as standard deviation (STD), peak, Kurtosis, etc. We also extract the Frequency domain statistics features [37,38], including mean frequency (MF), root-mean-square frequency (RMSF), standard deviation frequency (STDF), etc. We merge them as the final detection feature, and the feature descriptions are shown in the following Table 1.

**Figure 5:**
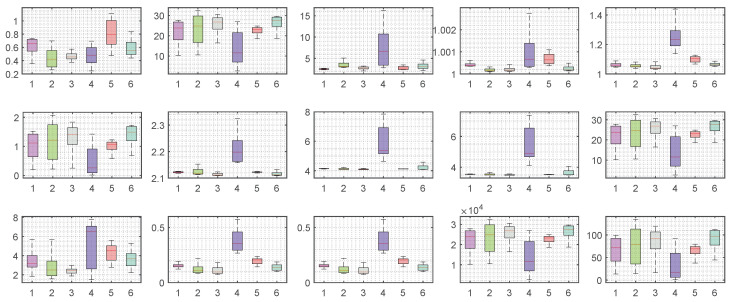
The box plots of the feature values. The X-axis is the different drinking and the Y-axis is the range of extracted feature values. Each subplot represents a box plot of the feature values of different drinking under one feature.

We plotted the box plots of the feature values, as shown in Figure 5. From Figure 5, we can see that the distribution of feature values of different drinking under each feature is different, which proves that our proposed features are effective to distinguish different drinking.

**Figure 6:**
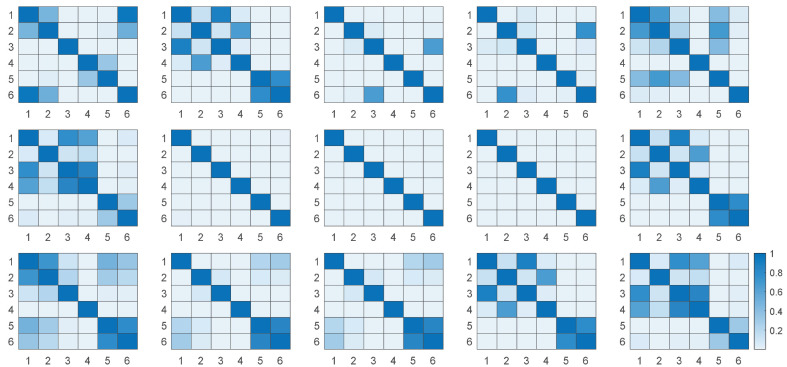
The heat maps of the featured F-tests. Each subplot is the F-test result for different drinking under one feature, and the rows and columns represent six drinking. The lighter the color, the smaller the F-test result; the greater the variability between the two drinking in the corresponding row and column, the better the feature.

To analyze the necessity of the 15 features, we have verified the effectiveness of the features based on the F-test, and the results of the F-test are represented in a heat map, as shown in Figure 6. Each subplot is the F-test result for different drinking under one feature, and the rows and columns represent six types of drinking. The smaller the F-test result, the better—that is, the lighter color proves that the difference between the two drinking in the corresponding rows and columns under that feature is greater and they can be distinguished more easily by this feature. As we can see from the figure, there are multiple areas with lighter colors in each subplot, which proves that the F-test results are significant between multiple drinking under our feature. It shows that all of our features can clearly distinguish between more than two types of drinking, proving the validity of our features. Therefore, we do not need to redundantly remove features.

### 3.3. Detection

**Figure 7:**
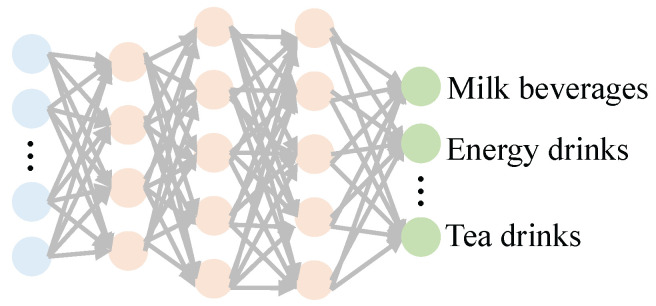
ANN-based drinking category detection model.

Our design uses a fully connected, feed-forward artificial neural network for drinking category detection, as shown in Figure 7. The input to the detection model is the features extracted above, and the output is the label of the drinking. Generally speaking, building and using a detection model are two steps, including training the model and using the model.

The extracted feature length determines the number of nodes within the input layer, and the drinking categories affect the number of nodes within the output layer. The detection model learns how to relate CSI measurements to different drinking categories based on the training data. Once the model has learned the mapping, it can easily be used to test the CSI measurements. We use the back propagation of the Stochastic Gradient Descent (SGD) method and the cross-entropy loss function to train the detection model, see Appendices Appendix A and Appendix B for details. The training cost of the model comes from two parts, including collecting and preprocessing training data, and building the detection model.

## 4. Experimental Results

### 4.1. Experimental Setup

**Figure 8:**
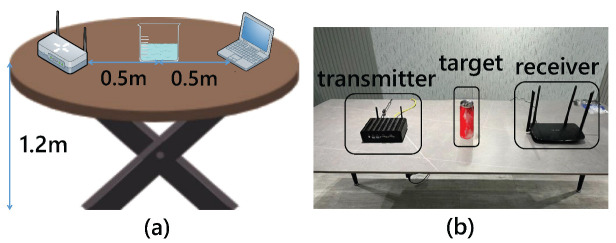
Experimental setup. (**a**) Device setup image. (**b**) Experimental environment image.

***Wireless devices setup.*** We employ a TL-WR886N router as the transmitter and an IWL 5300 NIC with a mini PC as the receiver to collect CSI. In our design, shown in Figure 8, we deploy the transmitter on one side and the receiver on the other side, the transmitter is 1 m from the receiver, and the drink to be tested is placed in the midline position between the two. Besides, the table is 1.2 m from the ground.

***Drinking categories.*** In our design, we chose six common drinks as test targets, including Coke, freshly squeezed watermelon juice, RedBull energy drink, black tea, milk, and instant coffee. All drinks were purchased from the supermarket; so, the density and materials of the same drinks to be tested were kept fixed. During the data collection, the volume of all drinks to be tested was fixed at 300 mL, and the collection environment and container were kept constant.

***Model Parameters.*** Our design utilizes sigmoid as the kernel function, the loss function is the Mean absolute error performance function (MAE), and the optimization problem is solved using Stochastic Gradient Descent (SGD).

***Model Evaluation.*** In drinking category detection model, we consider the detection performance impact of different hidden layer numbers starting from two to ten and different numbers of neurons in the hidden layer from 100 to 1000. In addition, we compare our detection model with current common learning methods and we use cross-validation to evaluate our design.

***Evaluation metrics.*** Four evaluation metrics, including accuracy, precision, recall, and f1-score, are used to evaluate the effectiveness of the proposed method in the experiment. When TP, TN, FP, and FN represent the true positive rate, the true negative rate, the false positive rate, and the false negative rate, respectively, the following equation may be used to calculate the above four evaluation metrics:

(3) $$accuracy=\frac{TP+TN}{TP+TN+FP+FN}$$

(4) $$precision=\frac{TP}{TP+FP}$$

(5) $$recall=\frac{TP}{TP+FN}$$

(6) $$f1-score=2\times \frac{precision\times recall}{precision+recall}.$$

### 4.2. Main Findings of the Evaluation

The main findings of evaluations are as follows:

- Our method achieves about 87.9% accuracy for detecting the drinking categories. The results show that this method can successfully achieve drinking category detection, which promotes its actual implementation in further development.
- Our system is novel and intelligent compared with current drinking category detection methods. The system’s novelty and intelligence are represented in the fact that it does not need any support of professional devices and it can be achieved using commercial devices. However, our design only provides a prototype framework; more drinking categories can be detected and additional intelligent functions can be developed in the future.

### 4.3. Overall Performance

**Figure 9:**
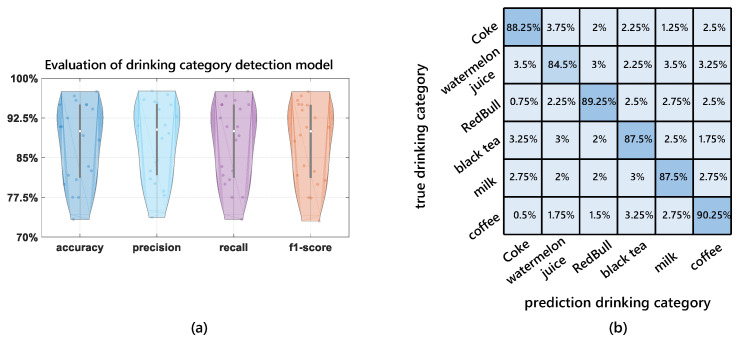
Overall performance. (**a**) Overall performance of evaluation metrics. (**b**) Overall performance of confusion matrix.

In all detection models, we fixed the training datasets and test datasets. Besides, we used the evaluation metrics above to evaluate the detection performance, which is shown in Figure 9a. Besides, we plot the confusion matrix for the performance of our design, as shown in Figure 9b. Note that in the experiment, we use a seven-layer ANN with 500 nodes to detect the drinking category.

Figure 9 shows the drinking category detection performance in the violin plot. It can be seen from Figure 9 that our design can detect the drinking category with an accuracy of 87.9%, and the average precision, recall, and f1-score of drinking detection are 88.3%, 87.9%, and 87.8%, respectively.

### 4.4. The Network Parameters

In the parameter adjustment of neural network, the number of hidden layer nodes and the number of layers are also used closely, which can fully adjust the effect of neural network. The activation function and model complexity control play a major role. In the process of neural network training, parameter adjustment is through continuous attempt and running, and it is a common optimization method to adjust the four parameters in the neural network model. These parameters need to be combined continuously to achieve the best model. The most typical parameters are the number of hidden layers, the number of nodes in each hidden layer, and the loss function. Thus, in the experiment, we evaluate the three parameters.

#### 4.4.1. Number of Hidden Layers

***The results show that a seven-layer ANN will be the better choice of our design.***

**Figure 10:**
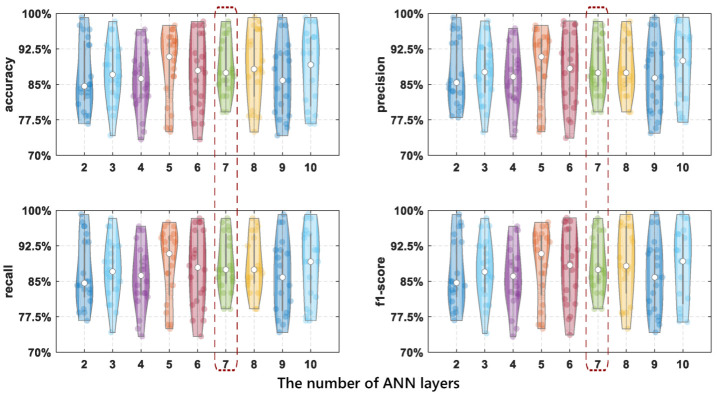
The comparison results of drinking category detection performance using different ANN layers.

More network layers can better help the network capture relationships, but can also lead to overfitting. Therefore, to evaluate the impact of hidden layers on the method performance, we increase the number of hidden layers from two layers to ten layers. The convolution kernel structure is the same in the experiment and the results of evaluation metrics are shown in Figure 10. The experimental results show that detection performs well when the ANN used is seven layers.

As can be seen from Table 2, the accuracy of eight layers is the highest, which is 88.79%; seven layers have the second highest accuracy of 88.62%; and the three-layer accuracy is the lowest, which is 86.04%. The difference between the seven-layer and eight-layer accuracy is about 0.1%, which can be ignored, and the difference from the lowest accuracy is about 3%. The more hidden layers, the higher the training time. Besides, from Figure 10, we can see that the seven-layer accuracy distribution is more compact; so, our design finally chooses seven layers as the most suitable number of hidden layers—of course, eight layers can also be chosen.

#### 4.4.2. Number of Neurons in Hidden Layer

***The results demonstrate that 500 is an excellent choice for ANN nodes at each layer.***

**Figure 11:**
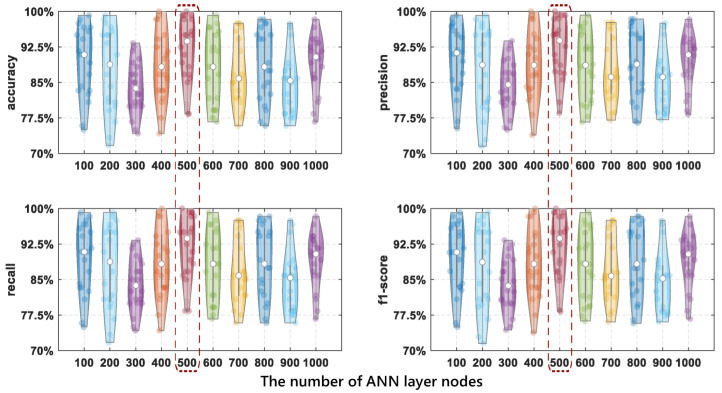
The comparison results of drinking category detection performance using different nodes of ANN layers.

When the number of nodes in the network is too large, the information processing ability is enhanced, causing the limited amount of data included in the training dataset to not be enough to train all the neurons in the hidden layer, and it is difficult to obtain the expected effect. In order to properly choose the number of nodes for each layer in ANN, we choose the number of nodes in each layer from 100 to 1000 to compare the performance improvements. It should be noted that the network structure employed in the models is the same. Figure 11 shows how the number of ANN layer nodes impacts the detection performance. As shown in Figure 11, when the number of nodes is 500, our design achieves pretty high accuracy in drinking category detection.

As can be seen from Table 3, when the number of nodes in ANN hidden layers is 500, the accuracy of the drinking category detection model is the best, which is 91.8%. When the number of nodes is 300, the accuracy of the detection model is the worst, about 84%. Compared with other node numbers, the difference between the highest and lowest accuracy is about 8%, which is about 4% higher than the average accuracy. Therefore, 500 nodes are the best choice, and the detection performance is the best at this time.

#### 4.4.3. The Different Loss Function

***Experimental results show that using MAE as loss function has higher detection accuracy.***

**Figure 12:**
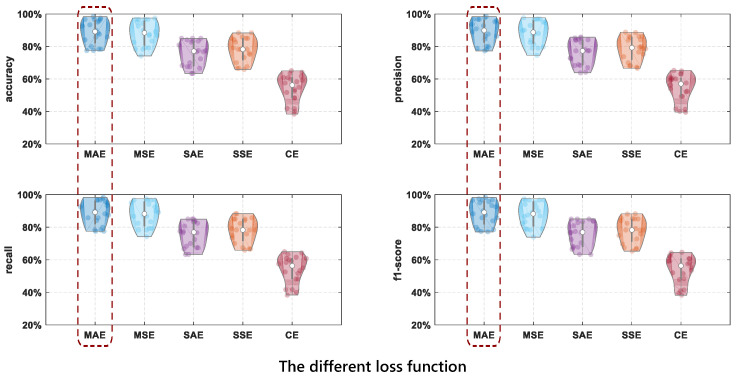
The comparison results of drinking category detection performance using different loss functions.

The loss function is a measure of the performance of the prediction model. No loss function can be applied to all types of data. In order to select the loss function suitable for the drinking category detection model, we selected the loss function including MAE, Mean squared error performance function (MSE), Sum absolute error performance function (SAE), Sum squared error performance function (SSE), and Cross-entropy performance function (CE) to compare their performance. The network structure used in the model is the same. Figure 12 shows how different loss functions affect the detection performance. As shown in Figure 12, when the loss function is MSE, the performance of drinking category detection is the best.

As can be seen from Table 4, when the loss function is MAE, the performance of the drinking category detection model is the best (88.8%) and the time complexity is good (2.8). When the loss function is MSE, the accuracy is the second best, which is 0.75% lower than the highest accuracy, but the time complexity is 2.5 higher than MAE. When the loss function is CE, the performance of the model is the worst (54.1%) and the time complexity is the best (1.3). Therefore, the loss function selected as MAE is the most suitable, with the best performance and good time complexity.

### 4.5. The Different Detection Models

***The results show that the ANN detection model has a significant performance improvement compared with other detection models.***

**Figure 13:**
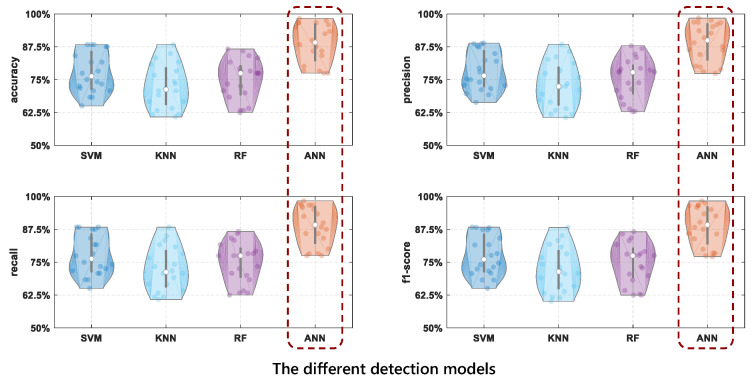
The comparison results of drinking category detection performance using different detection models.

To evaluate the drinking category detection performance, we compare ANN with three commonly used learning methods including SVM (Supported Vector machine), RF (Random Forest), and KNN (K-Nearest Neighbor). The results are shown in Figure 13. From Figure 13, we can see that ANN has the best detection performance compared with other algorithms.

From Table 5, we can see that the performance of traditional machine learning algorithms for drinking category detection does not vary much, with an average accuracy of about 75%. The ANN model has the best detection performance, higher than 88%, and the SVM is the next best, at about 78%. Therefore, the best performance for drinking category detection is achieved when ANN is chosen for the detection model.

## 5. Discussion

There is still potential for improvement in terms of the performance of our design, and we will discuss various points below.

**Feasibility.** Since our design requires a fixed transceiver, changes in device deployment may require the re-collection of fingerprints in real-world scenarios. We believe this will not be a problem as we can solve it using transfer learning. For the drinking category detection model used in this paper, in addition to the above six drinking categories, other types of drinking can also be added to the training dataset, allowing our design to identify more drinking categories. However, the increase in the variety of drinks may affect the detection performance;, we can extract other features that are better suited for drinking category detection, which is beyond the scope of this paper and will be the subject of future research.

**Depending on particular hardware cards.** In order to collect CSI measurements, specific NICs in the Linux system, which includes IWL 5300 NICs and Atheros NICs, must be used. However, both wireless transmitters and NICs are commercial devices, and they are quite inexpensive—for example, the IWL 5300 network card, costs around USD 3. With the growth of smart houses, wireless transmitters may become widespread. Furthermore, as CSI-based detection applications proliferate and mature, CSI will be accessible to upper layers via most NICs in the foreseeable future.

**Target.** The purpose of this paper is to detect drinking categories, and the drinking to be tested is a single category. We have not detected mixed drinking categories. Besides, there is research on adulterants identification based on wireless signals [12,14,47,48], and we have performed some validation experiments, but this is our future work.

**Impact factors.** Our design assumes no human activity in the current environment when detecting the drinking category, which is the assumption of most current wireless-signal-based detection methods. When the environment is noisy or there is human activity, the CSI measurements received at the receiver are mixed signals [24,27] of the target signals and environmental noise and they are difficult to separate. However, we believe that by combining the method of Wang [49] and Venkatnarayan [34], the noisy signals can be separated to improve the detection performance, which is our work in the future. Because the application scenario of our design is security check and self-checkout, all drinks are purchased from supermarkets, and the density and material of the same drinks to be tested are kept fixed. In addition, we designed experiments and found that the CSI measurements of different drinking in different equipment deployments, volumes, and container shapes are different and can be used as fingerprints to detect drinking categories. Furthermore, humidity has little impact on the propagation of WiFi signals [50]. In the actual application, we can re-collect data in a new environment to perform the detection.

## 6. Related Work

Material identification techniques play an important role in industry [51,52], technology, etc. For example, Zhou et al. [51] proposed a tool wear condition monitoring method in small samples. Dhekne et al. [14] distinguished between Diet Coke and Pepsi by UWB signals. Our work focuses on designing a novel drinking category detection method based on wireless signals and an artificial neural network. Current drinking category detection technologies are generally divided into four types: instrument-based methods, wireless-signal-based methods, sensor-based methods, and optical-based methods. We will introduce them as follows. Table 6 shows these methods and their differences from our design.

**Instrument-based methods.** This method utilizes the differences in chemical properties of different drinking, applies instruments to analyze them, and then detects the target [7,8,9,10]. For example, Agilent Technologies [10] uses the instrument to measure the permittivity of the target to be measured, and since different drinking has different permittivity, it can be used to detect drinking. However, this method requires that the chemical properties of the different drinking are significantly different; otherwise, the detection performance is significantly reduced. Moreover, the method with an instrument is contacting, which means that it can contaminate the drinking to be measured.

**Wireless-signal-based methods.** This method utilizes the propagation characteristics of wireless signals through the target drinking to detect the drinking category. Currently, there are several popular wireless-signal-based liquid detection methods: based on RF signals [11,12,13,53], based on UWB signals [14,54], radar-based signals [48,55]. For example, TagScan [12] detects liquids by extracting the Received Signal Strength Indicator (RSSI) and phase changes from the RF signals. However, this method requires a complicated setup and is time-consuming to label each target. LiquID [14] identifies liquids based on UWB signals by estimating the permittivity. However, UWB signals are not universal in daily life. FG-LiquID [48] identifies 30 different liquids based on Radar signals. However, radar devices are expensive and radar signals interfere with noise.

**Optical-based methods.** This method analyzes the optical spectra of different liquids by obtaining information about the optical absorption or reflection from the liquid to detect the target [56,57,58]. For example, Al-light [56] utilizes the principles of near-infrared spectroscopy to detect alcohol concentration. However, the method requires specialized equipment and professional people to operate it.

**Table 6:** Drinking category detection related work.

Models		Pros	Cons
**Instrument-based** **methods**	Equipment [7,8,9,10]	high accuracy	equipment maintenance drinking contaminate
**Wireless-signal-based** **methods**	RF [11,12,13,53]	estimate the horizontal cut images of targe	waste resources
	UWB [14,54]	identify a wide variety	not universal signals
	radar [48]	adulterants differentiation	affected by noise
**Optical-based** **methods**	Device [56,57,58]	fine-grained detection	specialized equipment professional people operate

## 7. Conclusions

This paper presents a novel drinking category detection method based on wireless signals and an artificial neural network, which identifies which category (i.e., Coke, tea, milk) contains the detected target. A convolution kernel is first used to extract features automatically; then, *ANN* is used to detect the target. A large number of experiments are performed to demonstrate the effectiveness of the method, including model parameters comparison and currently used model comparison. Experimental results demonstrate the effectiveness of our design, which can achieve about 88% accuracy in multiclass classification. We believe that combined with multicategory detection and considering more interference factors and more drinking categories in current drinking category detection systems, the system can be more intelligent, which is beyond the scope of this paper and will be our future work.

## Figures and Tables

**Table 1:** Time–frequency domain statistics feature interpretations.

ID	Interpretation
**STD** [37,38,39]	The standard deviation of CSI measurements. Calculate the square of the difference between the CSI measurements and their means, and then calculate the square root of its arithmetic mean.
**RMS** [37,40]	The root-mean-square of CSI measurements. Calculate the mean of the square sum of the CSI measurements and square it.
**KP** [36,37,38,41]	The Kurtosis of CSI measurements. Calculates the fourth central moment for the CSI measurements and is divided by the second central moment squared.
**SF** [37]	The form factor of CSI measurements. Calculates the ratio of the root-mean-square and rectified mean of the CSI measurements.
**CF** [37,40]	The crest factor of CSI measurements. Calculates the ratio of the maximum value and root-mean-square of the CSI measurements.
**MF** [37]	The mean frequency of CSI measurements. Calculate the frequency of CSI and calculate its mean.
**FC** [37]	The frequency center of CSI measurements. Calculate the frequency of CSI and calculate its median.
**RMSF** [37]	The root-mean-square frequency of CSI measurements. Calculate the frequency of CSI and calculate its RMS.
**STDF** [37]	The standard deviation frequency of CSI measurements. Calculate the frequency of CSI and calculate its STD.
**Xr** [42]	The denominator of clearance factor of CSI measurements. Calculate the square root of the absolute value of the CSI measurements; then, calculate its mean and square it.
**pk** [43]	The peak of CSI measurements. Calculate the difference between the maximum and minimum of the CSI measurements.
**I** [40,43]	The impulse factor of CSI measurements. Calculates the ratio of the peak and rectified mean of the CSI measurements.
**L** [43]	The clearance factor of CSI measurements. Calculates the ratio of the peak and Xr of the CSI measurements.
**E** [36,38,39,41]	The time domain energy of CSI measurements. Calculate the sum of absolute values of the CSI measurements.
**p** [44,45,46]	The frequency of CSI measurements. Calculate frequency using Power Spectral Density.

**Table 2:** The results of hidden layer size.

Hidden Layer Size	2	3	4	5	6	7	8	9	10
**accuracy**	0.8696	0.8742	0.8604	0.8841	0.8746	**0.8863**	0.8878	0.8604	0.8825
**precision**	0.8738	0.8776	0.8637	0.8842	0.8793	**0.8863**	0.8863	0.8639	0.8865
**recall**	0.8696	0.8742	0.8604	0.8841	0.8746	**0.8863**	0.8863	0.8604	0.8825
**f1-score**	0.8696	0.8737	0.8600	0.8842	0.8793	**0.8863**	0.8878	0.8604	0.8820

**Table 3:** The results of the number of neurons in each hidden layer.

Hidden Neuron Size	100	200	300	400	500	600	700	800	900	1000
**accuracy**	0.8938	0.8758	0.8404	0.8867	**0.9183**	0.8804	0.8725	0.8879	0.8454	0.8900
**precision**	0.8981	0.8752	0.8459	0.8888	**0.9219**	0.8839	0.8777	0.8921	0.8508	0.8942
**recall**	0.8938	0.8758	0.8404	0.8867	**0.9183**	0.8804	0.8725	0.8879	0.8454	0.8900
**f1-score**	0.8939	0.8752	0.8401	0.8861	**0.9181**	0.8797	0.8726	0.8877	0.8454	0.8900

**Table 4:** The results of the different loss functions.

Loss Function	MAE	MSE	SAE	SSE	CE
**accuracy**	**0.8879**	0.8804	0.7583	0.7796	0.5408
**precision**	**0.8913**	0.8852	0.7654	0.7858	0.5452
**recall**	**0.8879**	0.8804	0.7583	0.7796	0.5408
**f1-score**	**0.8877**	0.8800	0.7586	0.7788	0.5386
**time complexity**	2.8125	5.2656	6.0156	2.6406	**1.2969**

**Table 5:** The results of different detection models.

Detection Model	SVM	KNN	RF	ANN
**accuracy**	0.7767	0.7246	0.7492	**0.8879**
**precision**	0.7830	0.7289	0.7543	**0.8913**
**recall**	0.7767	0.7246	0.7492	**0.8879**
**f1-score**	0.7758	0.7230	0.7482	**0.8877**

## Data Availability

The data presented in this study are available on request from the corresponding author.

## Abbreviations

The following abbreviations are used in this manuscript:

NFC	Not From Concentrated
CSI	Channel State Information
ANN	Artificial Neural Network
RF	radio frequency
UWB	Ultra-wideband
COTS	commercial off-the-shelf
PCA	principal component analysis
RSS	received signal strength
NIC	Network Interface Card
STD	standard deviation
MF	mean frequency
RMSF	root-mean-square frequency
STDF	standard deviation frequency
SGD	Stochastic Gradient Descent
MAE	Mean absolute error performance function
MSE	Mean squared error performance function
SAE	Sum absolute error performance function
SSE	Sum squared error performance function
CE	Cross-entropy performance function

## Appendix A. ANN

Artificial neural networks (abbreviated ANN), also referred to as connection models or neural networks, are mathematical models of information processing that use a structure comparable to the synaptic connections of brain nerves. The neural network is simply to connect neurons to form a network, and it is characterized by multiple layers and full connection between neurons—that is, the neurons of the latter layer will be connected to each neuron of the former layer.

Generally, the input layer is the network’s leftmost layer, and the neurons in it are called the input neurons. The rightmost and output layers contain output neurons. The middle layer is known as the hidden layer because the neurons inside are neither input nor output. Neurons are the fundamental building blocks of neural networks, and there are multiple kinds of neurons. In this paper, we use sigmoid as the neurons and activation function of the network, which can be calculated as the following equation:

(A1) $$\sigma \left(z\right)=\frac{1}{1+e^{\left(-z\right)}}.$$

The neural network function is such that we provide it with a large amount of data (both input and output) for training in advance. After training, we hope it can also give a satisfactory output for the input of real environments. Therefore, we use the loss function, which can be represented by the difference between the real output and predicted output.

For a three-layer artificial neural network, it can be represented by the following equation:

(A2) $$f\left(x\right)=G(b^{\left(2\right)}+W^{\left(2\right)}\left(s\left(b^{\left(1\right)}+W^{\left(1\right)}x\right)\right)).$$

For the input layer, what you input is the input of input layer. For example, there are *n* neurons if the input is an *n*-dimensional vector. For the hidden layer, which is fully connected with the input layer, the output of the hidden layer is $f(w^{\left(1\right)}x+b^{\left(1\right)})$ while supposing that the input layer is presented by vector *x*, and $w^{\left(1\right)}$ is the weight (also known as the connection coefficient), $b^{\left(1\right)}$ is the offset, and *f* can be a commonly used tanh function or sigmoid function. The following equation may be used to calculate the tanh function:

(A3) $$tanh\left(z\right)=\frac{e^{z}-e^{-z}}{e^{2}+e^{-z}}.$$

Indeed, the hidden layer to the output layer may be considered as a multi-category logical regression—that is, softmax regression, the output of the output layer is $softmax(W^{\left(2\right)}x_{1}+b^{\left(2\right)})$, and $x_{1}$ represents the output $f(W^{\left(1\right)}x+b^{\left(1\right)})$ of the hidden layer. For a specific problem, how can we determine the connection weight and offset between layers? The gradient descent method (SGD) is used to solve the optimization problem, and it first randomly initializes all parameters, then trains iteratively, continually calculating gradients and updating parameters until the given condition is fulfilled, for example, when the error is small enough or the number of iterations is large enough.

The connection weights between nodes are adjusted according to the back-propagation error, and the correction direction of each weight is the opposite direction of the gradient of the error function. Let $w_{ij}$ be the weight of the ith hidden layer node to the jth output layer node, η be the learning rate, and *E* represent the error function of the output layer. We can obtain the following equation:

(A4) $$\Delta w_{ij}=-\eta \frac{\partial E}{\partial w_{ij}}.$$

## Appendix B. Loss Layer

Generally, the loss layer is used in the training phase of the model. After each batch of training data is sent to the model, the predicted result is output through forward propagation, and the loss layer calculates the difference between the predicted result and the real result. Then, the difference is used to update each parameter of the model through back propagation to reduce the loss between the real result and the predicted result, so that the predicted result generated by the model moves closer to the real result, so as to achieve the purpose of learning. The loss layer can be used to solve both regression and classification problems, which accomplishes this based on the loss function.

The loss function is an operation function used to measure the difference between the predicted result and the real result of the model, which is a non-negative, real-valued function. The smaller the loss function, the more robust the model will be.

Currently, the commonly used loss functions of ANN are MAE, MSE, SSE, and CE, which will be introduced as follows.

MAE is a common loss function used in regression models and classification models. It calculates the sum of the absolute values of the difference between the real result and the predicted result and represents the average margin of error of the predicted result, regardless of the direction of the error. MAE is applicable to the situation where there are outliers in the training data, and it can be updated in the direction of reducing the error of outliers through calculation without degrading the overall performance of the model. The formula of the MAE is expressed as

(A5) $$MAE=\frac{1}{n}\sum_{i=1}^{n}\left|f_{i}-y_{i}\right|$$

where $f_{i}$ is the output result of neural network and $y_{i}$ is the real result for each group of input, when there are assumed *n* groups of sample data including input and real results (also called expected results or expected outputs).

MSE calculates the Euclidean distance between the predicted result and the real result. In the classification problems, the ANN model converts the labels to calculate the loss between the predicted result and the real result. In regression problems, MSE is used to calculate the distance from the sample points to the regression curve, the sample points can better fit the regression curve by minimizing the squared loss, and its formula can be expressed as

(A6) $$MSE=\frac{1}{n}\sum_{i=1}^{n}{\left(f_{i}-y_{i}\right)}^{2}.$$

Absolute error refers to the difference between the predicted result and the true result, which reflects the magnitude of the deviation of the predicted value from the true value; so, it is called absolute error. SAE sums the absolute errors according to the following formula:

(A7) $$SAE=\sum_{i=1}^{n}\left|f_{i}-y_{i}\right|.$$

SSE calculates the sum of the squares of the error corresponding to the real result and the predicted result. After fitting an appropriate model according to *n* observations, the remaining part that cannot be fitted is called error; the sum of squares of all *n* residuals is called sum of squares of errors, and the formula is as follows:

(A8) $$SSE=\sum_{i=1}^{n}{\left(f_{i}-y_{i}\right)}^{2}.$$

CE is used to evaluate the difference between the predicted result obtained by training and the real result. In the classification problem of uneven positive and negative samples, CE is often used as the loss function because the surface of CE is very steep; so, the learning speed is relatively fast when the model effect is poor, which is conducive to the iteration of gradient descent. At present, CE is a commonly used classification loss function in neural networks, and can also be used to solve regression problems. It can be calculated as the following equation:

(A9) $$CE=-\sum_{i=1}^{n}f_{i}\log_{}y_{i}.$$
